# Use of Botanical Varieties of *Brassica oleracea* L. in the Breeding of Forage Kale

**DOI:** 10.3390/plants11162160

**Published:** 2022-08-20

**Authors:** John E. Bradshaw

**Affiliations:** Honorary Associate, James Hutton Institute, Dundee DD2 5DA, UK; johnbradshaw949@btinternet.com

**Keywords:** crude protein, digestible organic matter, dry-matter yield, plant height, S-methylcysteine sulphoxide, thiocyanate ion

## Abstract

At present, forage kale cultivars for feeding cattle and sheep are either open-pollinated ones from population-improvement schemes within suitable botanical varieties of *Brassica oleracea* or triple-cross hybrids from within or between botanical varieties, the only commercialised latter type being between marrow-stem kale and Brussels sprouts. Eight botanical varieties (15 cultivars) and 13 types of hybrids (50 hybrids) between them were produced and assessed for forage traits in SE Scotland in the early 1980s when there was government funding in Great Britain for such work (terminated in 1990). These previously unpublished results may now be of interest to a new generation of commercial forage brassica breeders. In addition to height and dry matter yield and content, quality traits, such as digestibility and antimetabolites, were assessed. The hybrids with marrow-stem kale as one parent varied in height, but combined a high-dry-matter yield with desirable quality traits for a forage crop. None was ideal and none had a superior combination of traits to the hybrids with Brussels sprouts. The hybrids between marrow-stem kale and January King cabbage were the shortest and a possible alternative to dwarf thousand-head kale. The results can be used to justify new forage brassica breeding programmes.

## 1. Introduction

Kale (*Brassica oleracea* L.) is grown as a forage crop in Great Britain (GB) and other countries with a similar climate [1,2]. Traditionally, the tall marrow-stem type was grown for dairy cattle in the autumn and the dwarf thousand-head type for sheep during the winter. These kales, along with the vegetables, broccoli, Brussels sprouts, cabbage, cauliflower, curled kale and kohlrabi, are all interfertile botanical varieties of *B. oleracea*, which can be used in forage brassica breeding [3]. The methods and types of cultivars are those appropriate for an insect-pollinated outbreeding species, in which self-pollination is largely prevented by a sporophytic incompatibility system [4], and forced inbreeding by bud-pollination results in lower yields [5,6,7]. Two types of breeding methods leading to two types of cultivars were developed in GB from 1951 to 1990 at government-funded breeding organisations.

The first method was population improvement leading to improved open-pollinated cultivars for ease of seed production. Examples are the population-improvement programmes at the former Scottish Plant Breeding Station (SPBS) [8] and former Scottish Crop Research Institute (SCRI) [9]. The starting population for the SPBS programme included Brussels sprouts, cabbages and curled kales in addition to marrow-stem and thousand-head kales. Four generations of selection for digestible organic-matter yield resulted in a population that consisted entirely of medium to tall marrow-stem kales, but with sufficient variation in leaf morphology to create uniformity problems in testing for distinctness, uniformity and stability during official National List Trials. Therefore, when a new programme started at SCRI with wider breeding objectives, the initial population was restricted to the 16 most clubroot-resistant marrow-stem kale cultivars available from a collection of 48 kale cultivars of diverse geographic origin. The result was two successful open-pollinated marrow-stem kale cultivars, Caledonian and Grampian, which were granted Plant Breeders’ Rights in 1996 and 2004, respectively, and are still being grown in 2022.

The second method was inbreeding and crossbreeding leading to double-cross [10,11] or triple-cross hybrids [1,12], F_1_ hybrids being ruled out because of the cost of producing large quantities of seed for agricultural use. The triple-cross hybrids from the former Plant Breeding Institute in Cambridge proved commercially successful and cultivars Maris Kestrel (released in 1967) and Bittern (1979) are still being grown in 2022. Maris Kestrel is a marrow-stem kale with a short, highly edible and digestible stem, which is moderately winter hardy. Bittern is a winter-hardy triple-cross hybrid between marrow-stem kale (M) and Brussels sprouts (B), made in such a way that the final cultivar is a hybrid between the two botanical varieties [{(M_1_ × M_2_) × M_3_} × {(B_1_ × B_2_) × B_3_}]. It combines the high-dry-matter yield of marrow-stem kale with the winter hardiness of Brussels sprouts [1]. Morphologically, it looks similar to marrow-stem kale with the leaves of Brussels sprouts. To the best of my knowledge, it is the only hybrid between botanical varieties that has been successfully commercialised.

Johnston [3], at the former Welsh Plant Breeding Station, produced double-cross hybrids of a number of botanical varieties (e.g., M and curled kale (C)) and analysed their performance (fresh-weight yield of leaf and stem) in terms of their constituent F_1′_s ((M_1_ × M_2_) × (C_1_ × C_2_), (M_1_ × C_1_) × (M_2_ × C_2_), (M_1_ × C_2_) × (M_2_ × C_1_)). Although consistent effects of order of combining the inbred lines on yield characteristics were not observed, a consideration of the differences in plant-to-plant uniformity showed that only the first combination was likely to be of practical value. Watts [13], in New Zealand, found that crosses between marrow-stem kale and both sprouting broccoli and cauliflower, and between wild cabbage and cauliflower, were the highest yielding (fresh-weight) of crosses between nine botanical varieties. Again, to the best of my knowledge, such double-cross hybrids have not been commercialised as forage crops.

To date, forage brassica breeding is performed by commercial companies, but they rely on the pre-1990 government-funded research for breeding methods as newer research is lacking. They need to decide which type of cultivar to produce and may wish to consider a hybrid between two botanical varieties of *B. oleracea.* This paper presents some previously unpublished work conducted at SCRI in which 8 botanical varieties (15 cultivars) and 13 types of hybrids (50 hybrids) between them were produced and assessed for forage traits in SE Scotland in 1982. It complements a recent paper on synthetic cultivar production by Bradshaw [7]. In addition to plant height (Height), dry-matter (DM) content and dry-matter (DM) yield, quality traits are considered: digestible organic matter in the dry matter (DOMD); crude-protein (CP) content; S-methylcysteine sulphoxide (SMCO); haemolytic anaemia factor, which can cause kale poisoning in ruminants [14,15]; and the thiocyanate ion (SCN^−^), a goitrogen released from the indolyl glucosinolates present in kale [16]. The results can be used to justify new forage brassica breeding programmes, which require pre-breeding to produce inbred lines from currently grown open-pollinated cultivars of marrow-stem kale, and also the assessment of forage traits of inbred lines of vegetable brassicas currently being used in commercial F_1_ hybrid breeding.

## 2. Results

Analyses were confined to the 65 trial entries where there were two cultivars of each botanical variety and a complete set of four hybrids, with the exception of winter cauliflower where there was just one cultivar and two hybrids (Table 1 and Table 2). There were statistically significant differences (*p* < 0.001) between the 21 groups of entries (8 botanical varieties plus 13 hybrids) when tested against the variation within the groups for all traits except DOMD% (*p* > 0.05). The variation within groups was also statistically significant (*p* < 0.01) when tested against the residual replicate × entries interaction.

The means of the 21 groups are shown in Table 2. Additionally, shown are those for the hybrids between the two tall marrow-stem kales (MS × MS), the two dwarf thousand-head kales (TH × TH) and the two curled kales (CK × CK). The standard error of difference (SED) between two means depended on the sizes of the groups, which are also shown in Table 2. Those shown in Table 2 are for the difference between two groups of sizes 2 and 4 (most botanical varieties and hybrids), and were calculated from the within group’s mean squares shown in Table 1. The marrow-stem kales and their hybrids with the dwarf thousand-head kales were the tallest groups, whereas the January King cabbages were the shortest group. The marrow-stem kales and their hybrids with the dwarf thousand-head kales and with the January King cabbages had the highest dry-matter yields, along with the hybrid between the two dwarf thousand-head kales. In contrast, the January King cabbages had the lowest yields. There was a wide range of dry-matter contents (percentages) from the kohlrabi group at 12.06% to the hybrid between the two curled kales at 21.46%. The differences in DOMD percentages were not statistically significant. There was quite a wide range of crude-protein (CP) contents from the hybrids between the Brussels sprouts and the marrow-stem kales at 12.59% to the January King cabbages at 21.64%. The curled kales, kohlrabies, and their hybrids, along with the January King cabbages, stood out as having SMCO contents greater than 9.5 g/kg DM. The hybrids between the Brussels sprouts and the marrow-stem kales had the lowest SMCO content of 5.45 g/kg DM. Finally, there was a wide range of thiocyanate ion content (SCN^-^) from the lowest in the marrow-stem kales at 35.0 mg/100 g DM to the highest in the January King cabbages at 96.2 mg/100 g DM, with the dwarf thousand-head and curled kales also having high contents.

Table 3 shows the comparisons of the means of the 13 hybrids (HY) with their mid-parent (MP) values. On average, the hybrids were taller (2 exceptions), had a higher dry-matter content and dry-matter yield, a slightly higher DOMD% (2 exceptions), but a lower CP% (1 exception), and lower levels of SMCO (1 exception) and SCN^-^ (2 exceptions). Furthermore, for all traits, there were hybrids that had higher or lower values than the parent with the highest or lowest value, and for DM%, CP% and SMCO the number of such hybrids was eight or more. The SEDs were calculated from the residual mean squares in Table 1 [½(MS)^½^] and could be used to compare a particular set of four hybrids (2 × 2) with their four parents (2 + 2), with the exception of WC × MS for which there was just one WC parent and hence two hybrids.

## 3. Discussion

The traditional types of forage kale grown in GB were the tall marrow-stem type for dairy cattle in the autumn and the dwarf thousand-head type for sheep during the winter. Two new types were bred in the 1960s and 1970s at the former Plant Breeding Institute in Cambridge [1,12]. Both resulting cultivars were triple-cross hybrids, which are still being grown. Maris Kestrel is a marrow-stem kale with a short, highly edible and digestible stem, which is moderately winter hardy, whereas Bittern is a winter-hardy hybrid between marrow-stem kale and Brussels sprouts. The main purpose of the research reported in this paper was to evaluate additional combinations of hybrids between botanical varieties of *B. oleracea*. The results should prove useful to forage kale breeders in deciding the type of kale to breed, despite being based on just one autumn trial with single-row plots. The growing season was fairly typical for SE Scotland with no abnormal stress conditions and hence good plant growth. However, the winter proved too mild to assess winter hardiness. Nevertheless, the high-dry-matter contents (>17%) of the curled kales, Brussels sprouts and their hybrids can be taken as indicators of winter hardiness.

The two tall marrow-stem kales, the two dwarf thousand-head kales and the two curled kales were included in an experiment conducted by Bradshaw and Borzucki [17] to assess the effect of harvest date on the chemical composition and digestibility of seven marrow-stem, three thousand-head and two curled kales from September 1979 to March 1980. They observed differences between cultivars and between harvest dates for all of the characteristics assessed, but cultivar by harvest date interactions were not a serious problem. All remaining plants in March were in very good condition as a result of the mild winter. The ranks of the six cultivars were the same for all six traits common to the two experiments; height not being measured by Bradshaw and Borzucki [17]. There were also differences between the three types of kale for all traits except DOMD%. In neither experiment did the ideal kale exist with respect to the nutritional characteristics examined, but the marrow-stem kales did have the highest dry-matter yields and lowest toxic-factor contents. In contrast, the culinary curled kales had low-dry-matter yields and high-toxic-factor contents, but also high-dry-matter and crude-protein contents. The thousand-head kales had values closer to the marrow-stem kales with the exception of their high thiocyanate ion content. The similarity of the results of the two experiments gives some confidence to proceeding to compare the hybrids with the three types of kale. Before doing so, it is also worth mentioning the results of Bradshaw and Borzucki [18] on the chemical composition and digestibility of 16 cultivars of horticultural cabbage, chosen to cover the range of types fed to livestock, and assessed over the period from 31 October 1979 to 15 February 1980. The cultivars did not differ significantly in DOMD%, which was high (82.9%), and the two January King cultivars had average dry-matter yields, but above-average dry-matter, crude-protein, SMCO and SCN^-^ contents. Hence, the two January King cultivars included in the current experiment are not typical of cabbages in general, but stand out as having the highest crude-protein, SMCO and SCN^-^ contents of the 21 groups in Table 2.

It is now time to discuss the potential of the 13 hybrids as forage crops. Apart from CP%, with few exceptions, the hybrids have a more favourable profile as a forage crop than the average of their parents, with a number being better than their better parent. This can be explained in terms of directional dominance for the desirable trait and differences in allele frequencies between the two parents [19], but need not concern us here. Taking cultivar Bittern as the established standard, Table 2 shows that the hybrids between the marrow-stem kales and the Brussels sprouts are slightly shorter than the tall marrow-stem kales and have a slightly lower dry-matter yield, higher dry-matter content, lower crude-protein content and similarly low SMCO and SCN^-^ contents. This is a good profile for a forage crop, apart from the crude-protein content, which was the lowest (12.6%) of the 13 hybrids. Watts [13] made pair-crosses between nine botanical varieties and reported the highest fresh-weight yields for those between marrow-stem kale and both sprouting broccoli and cauliflower. However, in the current study, these hybrids were not that different to the hybrid with Brussels sprouts. The hybrids with Brussels sprouts had a higher DM%, the ones with sprouting broccoli a higher dry-matter yield and the ones with cauliflower a higher DOMD%. Watts [13] also reported that the highest consumable yields, as assessed by feeding plants to sheep, also came from the hybrid with cauliflower together with the one between wild cabbage and cauliflower; a combination not tested in the current experiment. The hybrids between marrow-stem kale and January King cabbage are similar to the dwarf thousand-head kales in height, DM%, DOMD%, CP% and SMCO content, but have a higher dry-matter yield and lower SCN^−^ content, both desirable traits and a possible justification for considering such hybrids as an alternative to dwarf thousand-head kale.

The higher dry-matter yields and similar DOMD contents of the marrow-stem kales and their hybrids with other botanical varieties provide an explanation for the outcome of the SPBS population improvement programme [8]. Four generations of selection for digestible organic-matter yield resulted in a population that consisted entirely of medium to tall marrow-stem kales, having started with one, which also included Brussels sprouts, cabbages, curled kales and thousand-head kales [8]. Furthermore, the scores for transmission of pigmentation and curly leaves to offspring (see next section) explain the uniformity problems that can occur in an open-pollinated cultivar produced from such a population-improvement scheme. Hence, we reached the conclusion of Thompson [12] and Johnston [3] that the way to exploit variation between and within botanical varieties of *B. oleracea* in a forage crop is through double- or triple-cross hybrids in which the final cultivar is a hybrid between two botanical varieties.

Finally, we needed to consider what germplasm would be used today in new forage brassica breeding programmes aimed at a hybrid between a marrow-stem kale and a vegetable brassica. Suitable starting material could result from pre-breeding in which inbred lines are produced from currently grown open-pollinated cultivars of marrow-stem kale, and inbred lines of vegetable brassicas, currently being used in commercial F_1_ hybrid breeding, are assessed for forage traits. An example of a suitable open-pollinated marrow-stem kale as starting material is cultivar Grampian, released in 2004. It was the result of six generations of half-sib family selection for higher DM and DOMD contents, higher DOMD yield, lower levels of the antimetabolites SMCO and SCN^-^, medium height and clubroot resistance [9]. An old, but still relevant, example of inbred line production in vegetable brassicas is the work conducted at Birmingham University in the UK in the 1980s on Brussels sprouts [20]. They produced 2356 lines by single seed descent (SSD) and assessed them for both marketable yield and sprout quality. There is no reason why such lines could not be assessed for the traits appropriate for a forage crop.

## 4. Materials and Methods

### 4.1. Botanical Varieties

The 22 open-pollinated cultivars of the botanical varieties were as follows:

Tall marrow-stem kale (var. *acephala*): Giganta and Vulcan;

Dwarf thousand-head (var. *fruticosa*): Canson and Dwarf Thousand-Head;

Curled kale (var. *fimbriata*): Dwarf Green Curled and Tall Green Curled;

Sprouting broccoli (var. *italica*): White Sprouting Broccoli and Purple Sprouting Broccoli;

Kohlrabi (var. *gongylodes*): White Vienna and Purple Vienna;

Brussels sprouts (var. *gemmifera*): Vremo Inter and Bedford Winter Harvest;

Winter cauliflower (var. *botrytis*): English Winter Progress and Walcheren Winter-Birchington;

Cabbage (var. *capitata*): Copenhagen Market, Glory of Enkhuizen, January King 2, January King, Best of All, Ormskirk Rearguard, Holland Winter White E.40 and Asmer Late Winter Special.

### 4.2. Production of Hybrids between Botanical Varieties

During 1980, the six kale cultivars were crossed in all 15 combinations, and 63 out of a possible 96 combinations were achieved from attempts to cross the 6 kales to the other 16 cultivars. Plants were raised from seed sown during July 1979, the seedlings being pricked out into small poly-pots on 1 August and potted on into 12.5 cm pots the week beginning the 3 September. Then, from 16 to 24 October, 20 plants of each cultivar were potted on into 22.5 cm pots. They were kept in a frost-free glasshouse over winter to vernalize and started to flower at the beginning of April 1980. The emasculations and cross pollinations were performed by hand at the bud stage, using up to 10 plants of each cultivar, from 2 May to 20 June. Although the glasshouse was insect proof, as an additional precaution, the inflorescences were protected with Glassine bags for 2 weeks after pollination. The seed was harvested by the end of August.

### 4.3. Assessment of Hybrids

The trial site was the Murrays Farm, Pathhead, Midlothian, Scotland; the farm being a typical arable one in SE Scotland with the National Soil Map of Scotland showing a mineral gley soil. Agronomic practices were standard for leafy brassicas in SE Scotland, including applied fertiliser (176 N, 167 P_2_O_5_ and 167 K_2_O kg/ha) and insecticides to control cabbage root fly. The trial had a randomised complete block design with 2 replicates. The 100 entries (bought seed of 22 cultivars and 78 hybrids) were independently randomised within each replicate from the start of the experiment, on 26 and 27 April 1982, respectively, when 50 seeds in Petri dishes were placed in an incubator for 2 days at 20 °C to germinate. Then, 200 plots of 24 seedlings were raised in a glasshouse in Plantpaks containing a peat and sand compost. The 2 replicates were planted in the field and watered in on 2 and 3 June, respectively, when the plants were at the 3-leaf stage. Further water was applied on 7 June after a particularly hot day on 5 June (near 30 °C). The plots were now single rows of 12 plants spaced 50 cm within rows and 50 cm between rows. There were guard rows along the outside rows of the trial. Irrigation was not required during the growing season as there was adequate rainfall despite no rain between 18 and 31 July. The rainfall recorded at Edinburgh Airport, 15 miles away, was 81.77 mm in June, 41.92 mm in July, 49.80 mm in August, 88.13 mm in September and 81.80 mm in October. Average maximum and minimum temperatures in degrees centigrade for these five months were 16.5 and 9.5, 19.7 and 10.5, 18.6 and 10.4, 16.9 and 7.9, and 13.2 and 5.9, respectively. The extent of purple pigmentation (on a 1 to 9 scale of increasing pigmentation) and curliness of leaves (on a 1 to 9 scale of increasing curliness) were scored on 9 August.

Purple Vienna kohlrabi was completely purple in colour (score 9.0), as implied by its name, and transmitted this trait in varying degrees to its offspring (scores 4.5 to 7.5). Lower levels of pigmentation (scores ≤ 4.5) were also observed in some other hybrids, such as those between Tall Green Curled kale and both Dwarf Thousand-Head kale and Vremo Inter Brussels sprouts (scores 4.5). Dwarf Green Curled and Tall Green Curled kale (and their hybrid) had curly leaves (scores 9.0), as implied by their names, and transmitted this trait in varying degrees to their offspring (scores 3.5 to 7.0). No other cultivar or hybrid had curly leaves. Other aspects of leaf morphology were not recorded.

Plant height (Height) to the canopy top of each plot was measured on 14 September. Five plants (not end ones) from each plot were cut at ground level in the two replicates on 25 and 28 October, respectively. They were weighed for their fresh-weight yield, chopped, and two representative samples obtained. One was oven-dried for 20 h at 80 °C to determine its dry-matter (DM) content and the other was stored in a freezer at −20 °C until freeze-dried for chemical analyses. A final field inspection of the remaining plants occurred on 24 March 1983. The plants had all survived and were in good condition as a result of the mild winter. Hence, it was not possible to score winter hardiness.

### 4.4. Chemical Analyses

The oven-dried samples were milled through a 1 mm sieve in a hammer mill prior to analysis. Organic-matter content was determined by ashing in a muffle furnace. The digestible organic matter in the dry matter (DOMD) was determined using the cellulase method for brassicas as described by Allison and Borzucki [21].

Nitrogen (N) content was predicted on a fixed wavelength Technicon ‘InfraAlyzer’ [22]. A set of samples covering the range of predicted values was checked by the Kjeldahl method and used to make adjustments by linear regression. The N values were multiplied by 6.25 to estimate the crude protein (CP) content.

CP = 6.25 × (0.987 + 0.692 × N)

DOMD and CP contents were expressed as a percentage of the DM.

The freeze-dried samples were milled through a 1 mm sieve in a hammer mill and then stored in plastic jars with screw-on caps in a deep freeze until they were analysed for S-methylcysteine sulphoxide (SMCO) content by the method of Gosden [23], as modified by Griffiths [24], and for thiocyanate ion (SCN^−^) content using the automated procedure of Gosden [25].

### 4.5. Statistical Analyses

Analyses of variance were performed and the standard errors of the differences between two means (SED) of interest were estimated as [MS(1/N_1_ + 1/N_2_)]^½^, where MS is the mean square for the appropriate ‘error’ variation and N_1_ and N_2_ are the numbers of values contributing to each of the means being compared.

## 5. Conclusions

Seven out the 13 hybrids between botanical varieties of *Brassica oleracea* had marrow-stem kale as one parent. These varied in height but combined a high-dry-matter yield with desirable quality traits for a forage crop. None was ideal and none had a superior combination of traits to the already commercially established hybrid with Brussels sprouts. The hybrids between marrow-stem kale and January King cabbage were the shortest and a possible alternative to dwarf thousand-head kale. The results can be used to justify new forage brassica breeding programmes. Suitable starting material could result from pre-breeding in which inbred lines are produced from currently grown open-pollinated cultivars of marrow-stem kale, and inbred lines of vegetable brassicas, currently being used in commercial F_1_ hybrid breeding, are assessed for forage traits.

## Figures and Tables

**Table 1:** Mean squares from analysis of variance and levels of significance for sources of variation where groups are 8 botanical varieties plus 13 hybrids.

Variation	Degrees Freedom	Height	DM%	DM Yield	DOMD%	CP%	SMCO	SCN^-^
Between groups	20	857.95 ***	20.484 ***	0.57708 ***	9.119 NS	19.046 ***	15.633 ***	1176.6 ***
Within groups	44	73.72 ***	2.941 ***	0.08618 ***	14.582 ***	5.904 ***	3.944 **	310.7 ***
Residual	64	35.59	1.246	0.04730	7.646	1.922	2.217	164.0

NS: *p* > 0.05; ** *p* 0.01–0.001; *** *p* < 0.001.

**Table 2:** Means for 21 groups (8 botanical varieties plus 13 hybrids) plus those for the hybrids between the two tall marrow-stem kales (MS × MS), the two dwarf thousand-head kales (TH × TH) and the two curled kales (CK × CK) where the SED is for comparing two groups of sizes 2 and 4 (most botanical varieties and hybrids).

Groups	Number within Groups	Height cm	DM%	DM Yield kg/Plot	DOMD%	CP%	SMCO g/kg DM	SCN^−^ mg/100 g DM
MS	2	86.3	15.05	1.55	77.44	14.53	6.30	35.0
MS × MS	1	85.0	14.13	1.60	74.31	16.42	6.05	35.0
TH	2	68.8	16.25	1.01	81.09	14.18	7.67	85.0
TH × TH	1	80.0	18.44	1.54	76.13	13.48	7.35	92.5
CK	2	62.5	18.61	0.54	78.95	15.88	10.00	80.0
CK × CK	1	55.0	21.46	0.81	80.18	13.61	8.40	76.3
SB	2	65.0	14.60	1.06	78.97	14.63	7.67	77.5
KR	2	47.5	12.06	0.47	78.78	17.92	10.82	53.7
BS	2	57.5	17.09	0.73	81.87	14.62	7.93	68.1
WC	1	57.5	15.40	0.43	78.59	15.75	8.55	77.5
JK	2	38.8	12.74	0.37	79.98	21.64	12.10	96.2
TH × MS	4	87.5	16.43	1.43	79.88	13.61	7.38	46.4
CK × MS	4	80.6	17.84	1.23	78.18	13.84	6.61	45.0
SB × MS	4	78.1	15.72	1.37	79.42	13.55	5.54	43.1
KR × MS	4	70.6	16.18	1.18	80.45	14.18	6.95	51.2
BS × MS	4	77.5	18.29	1.21	80.34	12.59	5.45	50.6
WC × MS	2	76.3	16.56	1.31	83.33	13.06	5.68	52.5
JK × MS	4	68.8	15.56	1.48	80.73	14.66	7.10	48.7
CK × TH	4	76.9	18.83	1.11	80.21	13.79	6.74	55.6
KR × TH	4	65.6	15.52	0.99	79.51	16.29	8.35	69.4
BS × TH	4	69.4	18.47	0.98	82.02	13.66	6.29	64.4
SB × CK	4	67.5	18.42	0.97	79.95	13.30	6.72	51.9
KR × CK	4	50.0	17.86	0.81	80.40	15.30	9.60	49.8
BS × CK	4	60.0	19.38	0.90	80.68	13.32	7.01	58.7
SED		5.26	1.05	0.180	2.34	1.49	1.22	10.8

Abbreviations: MS: tall marrow-stem kale, TH: dwarf thousand-head, CK: curled kale, SB: sprouting broccoli, KR: kohlrabi, BS: Brussels sprouts, WC: winter cauliflower (Walcheren Winter-Birchington) and JK: January King cabbage.

**Table 3:** Comparison of means of groups of hybrids (HY) with their mid-parent (MP) values.

**Groups**	**Number within** **Groups**	**Height (cm)**					**DM (%)**				**DM Yield (kg/plot)**			
		**HY**			**MP**		**HY**		**MP**		**HY**		**MP**	
TH × MS	4	87.5 *			77.6		16.43 *		15.65		1.43		1.28	
CK × MS	4	80.6			74.4		17.84		16.83		1.23		1.05	
SB × MS	4	78.1			75.7		15.72 *		14.83		1.37		1.31	
KR × MS	4	70.6			66.9		16.18 *		13.56		1.18		1.01	
BS × MS	4	77.5			71.9		18.29 *		16.07		1.21		1.14	
WC × MS	2	76.3			71.9		16.56 *		15.23		1.31		0.99	
JK × MS	4	68.8			62.6		15.56 *		13.90		1.48		0.96	
CK × TH	4	76.9 *			65.7		18.83 *		17.43		1.11 *		0.78	
KR × TH	4	65.6			58.2		15.52		14.16		0.99		0.74	
BS × TH	4	69.4 *			63.2		18.47 *		16.67		0.98		0.87	
SB × CK	4	67.5 *			63.4		18.42		16.61		0.97 *		0.80	
KR × CK	4	50.0 #			55.0		17.86		15.34		0.81 *		0.51	
BS × CK	4	60.0			60.0		19.38 *		17.85		0.90 *		0.64	
SED	HY-MP	2.98					0.558				0.109			
Mean		71.5			66.7		17.31		15.70		1.15		0.93	
**Groups**	**DOMD (%)**			**CP (%)**				**SMCO (g/kg DM)**				**SCN^−^ (mg/100 g DM)**		
	**HY**		**MP**	**HY**		**MP**		**HY**		**MP**		**HY**		**MP**
TH × MS	79.88		79.27	13.61 *		14.36		7.38 #		6.99		46.4		60.0
CK × MS	78.18 #		78.20	13.84 *		15.21		6.61		8.15		45.0		57.5
SB × MS	79.42 *		78.21	13.55 *		14.58		5.54 *		6.99		43.1		56.3
KR × MS	80.45 *		78.11	14.18 *		16.23		6.95		8.56		51.2 #		44.4
BS × MS	80.34		79.66	12.59 *		14.58		5.45 *		7.12		50.6		51.6
WC × MS	83.33 *		78.02	13.06 *		15.14		5.68 *		7.43		52.5		56.3
JK × MS	80.73 *		78.71	14.66		18.09		7.10		9.20		48.7		65.6
CK × TH	80.21		80.02	13.79 *		15.03		6.74 *		8.84		55.6 *		82.5
KR × TH	79.51 #		79.94	16.29 #		16.05		8.35		9.25		69.4		69.4
BS × TH	82.02 *		81.48	13.66 *		14.40		6.29 *		7.80		64.4 *		76.6
SB × CK	79.95 *		78.96	13.30 *		15.26		6.72 *		8.84		51.9 *		78.8
KR × CK	80.40 *		78.87	15.30 *		16.90		9.60 *		10.41		49.8 *		66.9
BS × CK	80.68		80.41	13.32 *		15.25		7.01 *		8.97		58.7 *		74.1
SED	1.38			0.693				0.744				6.40		
Mean	80.39		79.22	13.93		15.47		6.88		8.35		52.9		64.6

* More extreme than parents, # atypically higher or lower than MP value. Abbreviations: MS: tall marrow-stem kale, TH: dwarf thousand-head, CK: curled kale, SB: sprouting broccoli, KR: kohlrabi, BS: Brussels sprouts, WC: winter cauliflower (Walcheren Winter-Birchington) and JK: January King cabbage.

## Data Availability

There was no requirement to publicly archive data when the analyses were done in 1984 and hence only the author has a printed copy of the original data.
